# Human Exposure to Novel *Bartonella* Species from Contact with Fruit Bats 

**DOI:** 10.3201/eid2412.181204

**Published:** 2018-12

**Authors:** Ying Bai, Modupe O.V. Osinubi, Lynn Osikowicz, Clifton McKee, Neil M. Vora, Maria Rosales Rizzo, Sergio Recuenco, Lora Davis, Mike Niezgoda, Ajoke M. Ehimiyein, Grace S.N. Kia, Akin Oyemakinde, Olufunmilayo Sanni Adeniyi, Yemi H. Gbadegesin, Olugbon A. Saliman, Abiodun Ogunniyi, Albert B. Ogunkoya, Michael Y. Kosoy, Idanre Bat Festival Investigation Team

**Affiliations:** Centers for Disease Control and Prevention, Fort Collins, Colorado, USA (Y. Bai, L. Osikowicz, C. McKee, M.R. Rizzo, M.Y. Kosoy);; Centers for Disease Control and Prevention, Atlanta, Georgia, USA (M.O.V. Osinubi, N.M. Vora, L. Davis, M. Niezgoda);; Colorado State University, Fort Collins (C. McKee);; Universidad Nacional Mayor de San Marcos, Lima, Peru (S. Recuenco);; Ahmadu Bello University, Zaria, Nigeria (A.M. Ehimiyein, G.S.N. Kia);; African Field Epidemiology Network, Abuja, Nigeria (A. Oyemakinde);; Federal Ministry of Health, Abuja (O.S. Adeniyi);; Federal Ministry of Science and Technology, Ibadan, Nigeria (Y.H. Gbadegesin);; Ministry of Agriculture and Natural Resources, Ilorin, Nigeria (O.A. Saliman);; Nigeria Center for Disease Control, Abuja (A. Ogunniyi);; Centre for Control and Prevention of Zoonoses/Rabies in West Africa International, Ibadan (A.B. Ogunkoya)

**Keywords:** *Bartonella*, bats, bat festival, bat flies, human, zoonoses, vector-borne infections, bacteria, Nigeria

## Abstract

Twice a year in southwestern Nigeria, during a traditional bat festival, community participants enter designated caves to capture bats, which are then consumed for food or traded. We investigated the presence of *Bartonella* species in Egyptian fruit bats (*Rousettus aegyptiacus*) and bat flies (*Eucampsipoda africana*) from these caves and assessed whether *Bartonella* infections had occurred in persons from the surrounding communities. Our results indicate that these bats and flies harbor *Bartonella* strains, which multilocus sequence typing indicated probably represent a novel *Bartonella* species, proposed as *Bartonella rousetti*. In serum from 8 of 204 persons, we detected antibodies to *B. rousetti* without cross-reactivity to other *Bartonella* species. This work suggests that bat-associated *Bartonella* strains might be capable of infecting humans.

Bats are natural reservoirs for a variety of pathogens (*1*). However, despite the risk to human health, persons around the world still intentionally handle bats, often without taking appropriate precautions. This lack of precautions is particularly evident in the tropics, where bats are abundant and frequently roost within or in close proximity to humans and domestic animals. In Asia and Africa, larger fruit bats (family *Pteropodidae*) are used as food, for either cultural reasons or subsistence (*2*). In some cultures, bat caves serve as spiritual sanctuaries (*3*).

One particular situation that has attracted the attention of scientists is a bat festival that takes place biannually in the Idanre Hills area of Nigeria. During the festival, which has occurred for many years, men enter designated caves, often without appropriate personal protective equipment, to capture bats. Local customs forbid persons from entering the caves outside of these festivities without permission from the community leadership. The captured bats are then eaten, used in cultural rituals, or sold as bushmeat (*3*). The predominant bat species within the caves is the Egyptian fruit bat (*Rousettus aegyptiacus*); colony sizes can reach >1,000 (*4*). Egyptian fruit bats are known reservoirs of zoonotic pathogens including Lagos bat virus, Marburg virus, and *Yersinia pseudotuberculosis* (*5*–*8*). Given the close human-to-bat contact that occurs during the festival, there is a risk for spillover of batborne pathogens to humans.

The genus *Bartonella* currently includes >30 species of bacteria (*9*), many of which have been described only recently. Various arthropod vectors seem to play an essential role in the maintenance and transmission of most known *Bartonella* species (*9*,*10*). In recent years, recognition of multiple *Bartonella* species as human pathogens responsible for a wide range of clinical manifestations has grown. Numerous novel strains of *Bartonella* have been discovered in bats of various species around the globe, including the human pathogen *Candidatus* Bartonella mayotimonensis, which was originally detected in aortic valve tissue of a person with endocarditis (*11*–*13*). In addition, a novel *Bartonella* genotype found in bats from the country of Georgia clustered with genotypes found in human forest workers from Poland (*14*). 

During 2010 and 2013, we researched the health risk to humans participating in the Idanre bat festival. We sampled bats and their ectoparasites from the caves and used them to identify a variety of zoonotic pathogens, including *Bartonella*. We recruited human participants from the surrounding community and surveyed them (through an orally administered questionnaire and serologic testing) to understand risk factors and the occurrence of pathogen spillover from bats to humans. We examined whether bats and ectoparasites of these bats within the caves used in the Idanre bat festival are infected with *Bartonella*, characterized any *Bartonella* species identified in bats or bat flies, and screened human serum samples for evidence of *Bartonella* infection.

## Materials and Methods

Human subjects work was approved by the Centers for Disease Control and Prevention (CDC) Institutional Review Board, the Ahmadu Bello University Human Ethics Board, and the National Health Research Ethics Committee of Nigeria. All animal procedures were conducted in compliance with a field protocol approved by the CDC Animal Institutional Care and Use Committee.

### Field Sites, Bat Capture, and Sample Collection

We captured bats by nets in 2 caves in Idanre Hills, Ondo State, southwestern Nigeria, in September 2010 (n = 106) and February 2013 (n = 71). We identified all bats by morphologic characteristics as Egyptian fruit bats (*R. aegyptiacus*). Captured bats were anesthetized by intramuscular injection of ketamine hydrochloride (0.05–0.1 mg/g bat weight) and exsanguinated via cardiac puncture after surface sterilization with 75% alcohol. Serum and blood clots were separated by centrifugation. Clots were stored at −80°C except while still in the field or being shipped, during which time they were stored on dry ice. 

### Bat Blood Culture and Characterization of *Bartonella* Strain

We plated bat blood clots on heart infusion agar containing 10% rabbit blood and incubated in an aerobic atmosphere with 5% carbon dioxide at 35°C for up to 4 weeks. Bacterial colonies morphologically identified as *Bartonella* were subcultured to obtain pure cultures.

We prepared crude genomic DNA by heating a heavy suspension of pure culture for 10 minutes at 95°C, followed by centrifugation of the lysed cells for 1 minute at 3,000 rpm. The supernatant was then transferred to a clean centrifuge tube to be used as the template DNA. We first verified all isolates obtained from the blood clots as *Bartonella* spp. by PCR amplification targeting a fragment of the citrate synthase gene (*gltA*) (*15*). Positive (*B. doshiae*) and negative (deionized water) controls were included to ensure that the PCR worked properly.

We purified and sequenced all PCR products of *gltA* in both directions by using an ABI 3130 Genetic Analyzer (Applied Biosystems, Foster City, CA, USA). We used the Lasergene software package (DNASTAR, Madison, WI, USA) to compare the generated *gltA* sequences with all available *Bartonella* species/genotypes. Once the sequences were identified, we selected 1 representative strain (R-191) for further characterization with multilocus sequence typing on the basis of sequence analysis of 8 molecular markers (*ftsZ*, *gltA*, *nuoG*, *ribC*, *rpoB*, *ssrA*, 16S rRNA, and internal transcribed spacers [ITS]) (*16*). For phylogenetic analyses, we used the neighbor-joining method by the Kimura 2-parameter distance method and bootstrap calculations with 1,000 replicates.

### Bat Ectoparasite Collection and Detection of *Bartonella* DNA

We collected ectoparasites from the skin and pelage of bats and stored them in microcentrifuge tubes with 70% ethanol. Ectoparasite species were identified by using available morphologic keys (*17*), and identifications were later confirmed by sequencing of the mitochondrial 16S rRNA and cytochrome oxidase I (COI) genes (*18*,*19*).

Using a Bullet Blender Gold homogenizer (Next Advance, Averill Park, NY, USA), we homogenized whole ectoparasites in Navy Eppendorf bead tubes (Next Advance) containing 400 μL brain–heart infusion broth (CDC, Atlanta, GA, USA). We extracted DNA from the homogenates by using the KingFisher Flex Purification System and the associated MagMAX Pathogen RNA/DNA Kit (both ThermoFisher, Waltham, MA, USA) according to the manufacturer’s protocols. Detection of *Bartonella* DNA in ectoparasite samples was performed by nested PCR for *gltA* (*20*) because of low concentrations of DNA and by conventional PCR for ITS (*21*), followed by sequencing and sequence analysis of amplicons.

### Preparation of Antigen from the *Bartonella* Strain Obtained from Bats

We produced a whole-cell antigen by co-cultivating Vero E6 cells with the pure culture (≈10^6^ agar-grown organisms) of the *Bartonella* strain (R-191) obtained from Egyptian fruit bats. Both were put into T-150 flasks that contained minimum essential medium supplemented with 10% fetal calf serum, 10 mmol HEPES buffer solution, 10 mmol nonessential amino acids, and 2 mmol L-glutamine. The flasks were incubated at 35°C and harvested on postinoculation day 4. At harvest, all but 2 mL of the medium was removed from the flask, sterile glass beads were added, and the flask was gently rocked to remove the Vero E6 cell monolayer. Drops (≈15 μL) of the cell suspension were mounted on each well of 12-well glass slides, which were then air dried, fixed in acetone for 15 minutes, and stored at −70°C until use.

### Human Serum Collection and Testing for Antibodies 

Persons in communities surrounding the caves who gave consent were enrolled in the study 11–15 days after the first bat festival of 2013 (February 19, 2013); not all of these persons had participated in all bat festival activities. Participants were asked about their contact with bats and their role in the festival, and some provided a blood sample (considered an acute-phase specimen). About 69–78 days later, a follow-up survey was conducted and a second blood sample (considered a convalescent-phase specimen) was collected (the second bat festival of 2013 did not take place between collection of the acute- and convalescent-phase samples). Serum and blood clots were separated by centrifugation; serum was stored at −80°C except while in the field or being shipped, during which time it was stored on dry ice.

To screen human serum, we used an indirect immunofluorescence assay at an initial dilution of 1:32 for IgG against the specific *Bartonella* antigen from the bat-associated isolate. Antigen-covered wells of the slide were overlaid with dilutions of human serum. Separate slides were included with positive and negative controls. The positive control against the Egyptian fruit bat–associated *Bartonella* species was produced in laboratory mice via mouse immunization with heat-inactivated bacterium (ProSci Incorporated, Poway, CA, USA). All slides were incubated at 35°C for 30 minutes and then washed in phosphate-buffered saline for 15 minutes. We used anti-human and anti-mouse conjugates (Kirkegaard & Perry Laboratories Inc., Gaithersburg, MD, USA) for human and control serum samples, respectively. Each human serum sample reactive at the initial dilution was further titrated in 2-fold dilutions to endpoint; to check for cross-reactivity, we tested the final positive samples (defined as a titer >1:64) for 3 other *Bartonella* antigens (*B. elizabethae, B. henselae,* and *B. quintana*) previously reported in Africa (*22*–*24*).

## Results

### *Bartonella* in Egyptian Fruit Bats

We recovered *Bartonella* isolates from 22 of 177 Egyptian fruit bat blood clots, giving an overall prevalence of 12.4%. The *gltA* sequences of all *Bartonella* strains obtained from Egyptian fruit bats were identical or similar (>97% identity) to each other and represented 4 unique variants (GenBank accession nos. HM363764, MH069693–MH069695). A variant is defined when it differs by >1 nt from others. Together with *Bartonella* strains obtained from Egyptian fruit bats in Kenya (*25*), these variants constitute a monophyletic genogroup that is distant from all other genotypes previously found in other bat species and any other described *Bartonella* species.

Multilocus sequence typing of the type strain (*gltA*; GenBank accession no. HM363764) with 7 additional genetic loci (*ftsZ*, *nuoG*, *ribC*, *rpoB*, *ssrA*, 16S rRNA, and ITS) further confirmed the uniqueness of this strain. Sequencing information for each genetic marker demonstrated that the *Bartonella* strain from the Egyptian fruit bats was distant from all other known *Bartonella* species and genotypes, including those reported from other bats from Africa. Sequences of all genetic loci obtained during the analyses were deposited in GenBank (accession nos. HM363769, KM387321, HM363779, HM363774, KM382247, HM363784, and KM382255). We compared the fragment sequences of each target with those from other *Bartonella* species/genotypes. The Egyptian fruit bat–associated *Bartonella* formed a separate genetic group that was distant from all other *Bartonella* species with >20% genetic distance and probably represents a novel *Bartonella* species, according to the definition of La Scola et al. (*26*). We proposed that this bacterial species be named *Bartonella rousetti*, to reflect the Egyptian fruit bat (*Rousettus aegyptiacus*) as the natural host. A phylogenetic tree based on the ITS locus illustrates the relationship of this proposed novel species to other *Bartonella* species (Figure).

**Figure:**
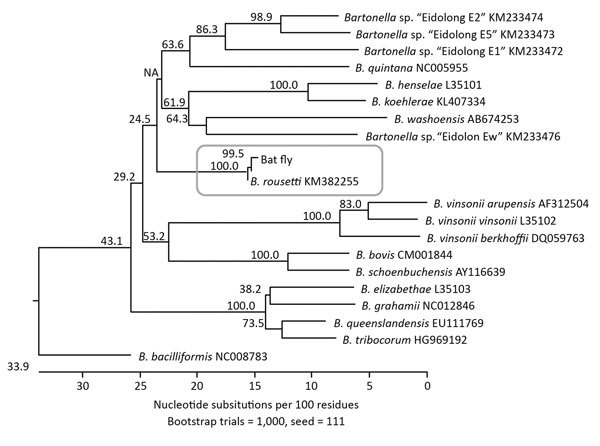
Phylogenetic relationships of *Bartonella rousetti* (proposed name) obtained from Egyptian fruit bats (*Rousettus aegyptiacus*) collected in Nigeria, 2010 and 2013, and other *Bartonella* species and bat-associated *Bartonella* based on internal transcribed spacer sequences. The neighbor-joining method by the Kimura 2-parameter distance method and bootstrap calculation was conducted with 1,000 replicates for phylogenetic analysis. The internal transcribed spacer sequence obtained from the bat flies was closely clustered with *B. rousetti*. GenBank accession numbers are provided for the *B. rousetti* sequence and the comparison sequences.

### Identification of Bat Flies and Detection of *Bartonella* DNA

In 2013, we collected 51 ectoparasites from Egyptian fruit bats. With the exception of 1 unidentified mite, all arthropods were identified as the bat fly *Eucampsipoda africana* Theodor (Diptera: Nycteribiidae). The morphologic identification of every bat fly was confirmed by 1 or both mitochondrial markers (16S rRNA or COI). Representative 16S rRNA (accession nos. MH138030–MH138037) and COI (accession nos. MH151059–MH151066) sequences have been deposited in GenBank.

Of the 50 DNA extracts from bat flies, 21 (42%) produced >1 ITS or *gltA* sequence that was confirmed via BLAST (https://blast.ncbi.nlm.nih.gov/Blast.cgi) as *Bartonella*. Positive samples yielded 19 ITS sequences and 18 *gltA* sequences; 16 samples yielded sequences for both loci and 5 samples yielded only 1 sequence. All but 1 of the 19 ITS sequences matched closely to the proposed *B. rousetti* (>95% sequence identity) (Figure); the remaining sequence was identical to *B. tamiae* (DQ395180). Of the 18 (66.7%) *gltA* sequences, 12 were close matches for *B. rousetti* (>98.3% sequence identity); all 12 of these samples also produced ITS sequences matching this strain.

The remaining 6 *gltA* sequences were identical to *Bartonella* sequences detected in a louse (*Neohaematopinus sciuri*) collected from a dead Eastern gray squirrel (*Sciurus carolinensis*) at a zoo in Greenville, SC, USA (GenBank accession no. EU368000) and an unidentified tick collected from a sheep in Peru (GenBank accession no. AF415209). These sequences were also closely (>99% sequence identity) related to other sequences from fleas (*Ctenocephalides felis* and *C. canis*) collected from dogs in Tunisia (GenBank accession nos. KP126468–74), a louse pool (*Polyplax* spp. and *Hoplopleura* spp.) collected from rodents in Thailand (GenBank accession no. KT324560), and an unidentified flea collected from a dog in Peru (GenBank accession no. GU583843). Of the specimens with this particular *gltA* sequence, 2 yielded no ITS sequence, 3 yielded ITS sequences matching *B. rousetti*, and 1 yielded the single *B. tamiae* sequence. Representative ITS (GenBank accession nos. MH14262–MH142639) and *gltA* (GenBank accession nos. MH151067–79) sequences for each novel sequence variant were submitted to GenBank.

### Human Exposure to *B. rousetti*

A total of 305 serum samples from 204 participants were tested for IgG against *B. rousetti*; 12 samples from different persons showed reactivity at an initial dilution of 1:32. Further 2-fold titration confirmed that 8 were positive, with titers >1:64 (Table). The positive samples were retested for 3 other *Bartonella* species—*B. henselae, B. quintana,* and *B. elizabethae*, all of which have been reported in Africa (*22*–*24*); antibodies against these *Bartonella* species were not detected in any of the samples. Five seropositive participants reported having eaten bats and having either touched bats or been scratched or bitten by them, although not all reported having ever participated in the bat festival. Three seropositive participants reported never having eaten bats, touched bats, or been scratched or bitten by bats; in addition, these 3 participants claimed to have never participated in the bat festival. Of the 8 seropositive participants, only 1 reported having experienced a febrile illness since the bat festival that had taken place earlier in the year.

**Table:** Epidemiologic data for persons with antibodies to *Bartonella rousetti* detected in study of human exposure to a novel *Bartonella* species from contact with fruit bats, Nigeria, 2013*

Participant age, y/sex	Titer in acute-phase serum†	Titer in convalescent-phase serum†	Ever ate bat	Ever participated in bat festival	Last time touched, scratched, or bitten by bat	Febrile illness since first bat festival of 2013
45/F	<1:32	1:64	Yes	No	6–12 mo ago	No
37/M	<1:32	1:64	Yes	No	>12 mo ago	No
25/F	<1:32	1:512	Yes	Yes	<1 mo ago	No
30/F	<1:32	1:512	No	No	Never	No
21/M	1:64	<1:32	No	No	Never	No
44/F	1:64	<1:32	Yes	Yes	<1 mo ago	No
70/M	1:256	No sample	Yes	No	>12 mo ago	Yes
32/F	1:256	No sample	No	No	Never	No

*Bartonella rousetti is the proposed name for the novel *Bartonella* species identified in Egyptian fruit bats in Nigeria. †Acute-phase samples collected within 11–15 d after first bat festival of 2013; convalescent-phase samples collected 69–78 d after acute-phase sample collection (the second bat festival of 2013 did not take place between collections of acute- and convalescent-phase samples).

## Discussion

We made several observations during this investigation. First, Egyptian fruit bats carry a unique *Bartonella* strain that probably represents a new species, for which we propose the name *Bartonella rousetti*. Second, bat flies, the common ectoparasites of bats, carry this same strain of *Bartonella*. Because this organism was detected by PCR only, the presence of the DNA does not necessarily indicate that the organism is viable. Last, persons from the communities surrounding the bat caves were exposed to this particular *Bartonella* strain, which might cause human infection.

Since 2010, several reports have described finding diverse *Bartonella* genotypes in bats of many species (*25*,*27*–*30*). The relationships between *Bartonella* genotypes and bat species that harbored these bacteria are not always simple. The same *Bartonella* species may circulate among different bat species, showing no specific relationship between the bats and the *Bartonella* species (*27*). Sometimes, multiple *Bartonella* species are associated with bats of only 1 species. For example, 6 *Bartonella* species have been identified in straw-colored fruit bats (*Eidolon helvum*) in Africa (*16*,*29*). Our study indicates that Egyptian fruit bats carry a specific *Bartonella* strain that has not been identified in bats of other species. 

Similarly, we found that the most prevalent *Bartonella* species found in bat flies parasitizing Egyptian fruit bats is *B. rousetti*. The ectoparasite bat flies *E. africana* are predominantly associated with Egyptian fruit bats (*17*,*31*,*32*). Although sequences matching other *Bartonella* species were identified in the bat flies, these genogroups may be primarily associated with arthropods and not mammals. One sequence from a bat fly was identified as *B. tamiae.* The presence of *B. tamiae* in bat flies from Algeria has been recorded (*33*), and the bacterium reportedly has been identified in chigger mites collected from rodents in Thailand (*34*). It is possible that *Bartonella* species found only in arthropods and not their associated mammal hosts may represent facultative symbionts that are uniquely adapted to live in the arthropod gut or other body system (*35*,*36*). The risks posed to humans by these primarily arthropod-associated *Bartonella* species are still unclear, although *B. tamiae* is a reported human pathogen that may cause febrile illness and other clinical signs and symptoms (*37*).

Detection of antibodies against *B. rousetti* in serum samples from several study participants indicates their exposure to the bacteria. However, with serologic results, cross-reactivity is a concern. For example, phylogenetically closely related *B. henselae* and *B. quintana* (the causative agents of cat-scratch disease and trench fever, respectively) exhibit a high level of serologic cross-reactivity (*26*,*38*,*39*). We tested the positive human serum samples for 3 other *Bartonella* species (*B. henselae*, *B. quintana*, and *B. elizabethae*) that circulate in Africa, and we did not detect any positive results. Given that immunofluorescence assays have good discriminatory ability for a wide range of antigens (*40*–*42*), the results lead us to conclude that the antibodies in these participants were indeed reactive with *B. rousetti* but not the other *Bartonella* species tested, although cross-reactivity with other non-*Bartonella* agents cannot be ruled out. 

Our study is not the first attempt to identify antibodies against bat-associated *Bartonella* in humans. Mannerings et al. (*43*) conducted a serologic survey of 335 volunteers from Ghana for antibodies against 6 species of *Bartonella*, including *Bartonella* strains isolated from straw-colored fruit bats. In that study, only 2 serum samples were positive for *B. henselae* antibodies at low titers, whereas none was positive against the bat strains.

All known species of *Bartonella* are transmitted between natural animal hosts by arthropods (*29*,*44*). The presence of *B. rousetti* DNA in *E. africana* bat flies parasitizing Egyptian fruit bats suggests that these ectoparasites may act as vectors for the transmission of *Bartonella* infection among bats, but it is unclear how bat flies would play a role in transmitting the bacterium to humans because bat flies do not commonly bite humans (C. McKee, unpub. data). Instead, human exposure may potentially occur via other routes, such as 1) directly by bat bites or scratches, which is similar to how humans acquire infections with *B. henselae* through cat scratches (*10*,*45*); 2) indirectly by contamination of open wounds with blood or other materials (e.g., saliva, urine, feces) of infected bats; or 3) indirectly by contamination of open wounds with bat fly excreta. Several studies have reported detecting *Bartonella* DNA in bat feces (*12*,*46*,*47*), and Dietrich et al. (*47*) detected *Bartonella* DNA in bat saliva and urine, providing support for routes 1 and 2 above, although no attempts have been made to culture viable bacteria from these fluids. However, viable *Bartonella* bacteria have been cultured from experimentally infected ectoparasites, including fleas and bedbugs (*48*,*49*), although such studies have yet to be performed for bat flies. Nevertheless, evidence is accumulating that *Bartonella* could spread from infected mammalian hosts through multiple routes. Therefore, it may not be necessary for humans to interact directly with live bats to be exposed to bat-associated *Bartonella*. Persons might be at risk when interacting with bat carcasses, guano, or other contaminated products. Of note, we do not provide definitive evidence of the route of exposure for any of the 8 seropositive participants. Indeed, 3 of these participants reported no interactions at all with bats.

Future studies should continue to evaluate the relative correlations of exposure routes, the pathobiology of batborne *B. rousetti* in humans, and vector competency of bat flies for transmitting *Bartonella.* Results should provide guidance to communities for mitigating the risks to humans interacting with animals and their arthropod vectors.
